# Looking after our menopausal workforce: A model for NHS staff

**DOI:** 10.1177/20533691221128352

**Published:** 2022-09-27

**Authors:** Janice Rymer, Debra Holloway, Deborah Bruce, Joan Bowen

**Affiliations:** 1 King’s College London, London, UK; 2 Guy’s and St Thomas’ Hospitals NHS Trust, London, UK

**Keywords:** Access to menopause care, NHS staff, staff menopause clinic

## Abstract

**Objective:**

To determine whether a staff menopause clinic would benefit our peri/postmenopausal hospital workforce.

**Methods:**

The three menopause consultants, with OH administration assistance, set up a virtual clinic for staff members to address the unanswered need for access to good menopause care. Feedback was gathered from the attendees and the staff who ran the clinic.

**Results:**

The clinic was an overwhelming success and has now become an established clinic at a major London teaching hospital.

**Conclusions:**

We hope that this model can be used in other trusts, and other companies to provide good menopause care to our senior female workforce to retain them. Acknowledgements: We would like to thank the OH staff who ensured the clinics ran so smoothly.

## Introduction

In the NHS workforce nearly 50% of the working population are women in the 45–65 age group. Currently many women are leaving or considering leaving the NHS because of menopausal symptoms and/or Covid consequences. Access to HRT for women was already difficult before the pandemic but now this is even more challenging and many General Practices are reluctant to prescribe HRT. Women take HRT to relieve their symptoms of estrogen deficiency and to protect against the long term effects of ovarian failure eg. osteoporosis, cardiovascular disease, Parkinson’s disease, dementia etc. NHS England/Improvement are currently rolling out a programme to retain women in the NHS workforce by providing information on the optimal pathways for menopausal care.

As clinicians running the Guy’s and St Thomas’s NHS Hospital Trust (GSTT) menopause clinic we were very aware of peri/postmenopausal colleagues who were retiring early and the resultant significant loss to the workforce. We therefore felt that we would like to contribute to the NHSE/I programme by giving our GSTT staff access to menopausal experts to discuss their individual situations and give advice. We had already done large group information meetings to staff and managers, (male and female) but many women were not happy to divulge their personal situation to others and preferred a one-to-one consultation. We had regular monthly webinars on women’s health issues that were well attended and evaluated, and the evaluations suggested a one to one clinic would be helpful for the staff in the Trust.

Discussions around menopause can normally occur in primary care but many practices are not comfortable to prescribe HRT without expert advice and particularly now, it is challenging for a postmenopausal woman to get an appointment in primary care for a non-acute, or non “red flag” problem. We therefore set up menopause clinics purely for GSTT staff to attend, with the hope that if this model worked, it could be adopted by other trusts.

## Aims

To provide a holistic consultation for staff members who are suffering from perimenopausal or menopausal symptoms within our trust and offer them a chance to speak to menopause experts with the aim of improving their health and wellbeing so they do not leave the NHS workforce, particularly at this challenging time.

This was also an ideal time to assess lifestyle and risk factors for future health.

We also wanted to assess the demand for these staff clinics and subsequent satisfaction with appointments

The original plan of NHSE/I was to target nursing staff as they are the female majority but we welcomed any female staff whatever job or location they had at GSTT. By making the clinics virtual we were able to cover all the areas including community staff.

## Methodology

The pilot menopause clinic was advertised through a variety of mediums including the Trust Web site, Staff Bulletin, email, Local Newsletters and via Managers. We did this with the administrative help of GSTT Occupational Health. Once they were given an appointment, they were asked to do a pre appointment questionnaire (Table 1) and then offered a 15 min virtual appointment.

**Table 1.** Staff Menopause clinic pre appointment questionnaire.

Symptoms causing referral	—	How long for
Date of last menstrual period	How often are your periods	How many days do you bleed
Are your periods a problem?	Have you have any unusual bleeding? Yes no	Have you had your womb removed?
	Details	
Are you taking HRT? Yes no	Name of HRT How long have	you been on this?
Have you any problems on this?	Have you taken any other HRTs and when And what ones	Have you had any problems with previous HRT’s
Have you taken any alternative menopause treatments	Are you taking any medications? Yes no if yes, which ones	When was your last cervical screening date And the result Normal abnormal
Have you had a mammogram? Yes no What was the result? …………………..	Have you had a bone scan? Yes no	What was the result Do you use contraception? Yes no what
What questions would you like answered?	—	

The clinics were done by us (JR,DH,DB) in our own time in the evenings with 15 min appointments, At the end of the appointment we would return the originally submitted form with our advice, to the woman and OH. These records were stored in OH. We advised but did not prescribe, so the woman could take the form to their GP with documentation from a menopause specialist.

If the GP was not happy to prescribe HRT then they could be referred to the menopause clinic, and if their situation was complex (comorbidities) then we would advise referral to the menopause clinic. We had agreement that anything urgent/red flag picked up such as post-menopausal bleeding could be referred straight into the 2 Week Wait service. Following the appointment, the attendee staff members were asked to complete an anonymous survey to provide feedback about their experience. The staff running the clinic were also asked to fill in a questionnaire.

## Results

At the booking stage, the clinic slots were taken up very quickly and there was a long waiting list of women who could not be accommodated. The demographics of the staff attendees are shown in Table 2. There was an 89% attendance rate and a 73% survey response rate. Data from the surveys shows 100% of the participants reported that their questions were answered by the clinicians, 100% were given a clear management plan and 100% said they would use the service again. On a scale of 0–10, participants rated an average of 8.6 in terms of how useful they found the clinics and an average of 8.9 said they would recommend these clinics to other staff members. Satisfaction Comments – Table 3.

**Table 2.** Demographics: Staff Role Band.

1	2	3	4	5	6	7	8a	8b	8c	8d	9	Other
0 0(%)	1 2(%)	6 11(%)	6 11(%)	4 7(%)	7 13(%)	13 24(%)	10 19(%)	0 0(%)	4 7(%)	1 2(%)	0 0(%)	2 4(%)

Ethnicity							
Black African Caribbean	Asian indian pakistani Bangladeshi Chinese japanese	White English Welsh Scottish N.irish	Irish	Romani or Irish Traveller	White Other	Arab	Mixed/Multiple Ethnicity
14 26(%)	2 4(%)	27 50(%)	2 4(%)	3 6(%)	3 6(%)	1 2(%)	2 4(%)

Staff Age Bands						
36-40	41-45	46-50	51-55	56-60	61-65	65+
0 0(%)	8 15(%)	18 33(%)	21 38(%)	6 11(%)	1 2(%)	0 0(%)

**Table 3.** Staff attendees free text comments.

• The clinician was completely understanding and gave great advice.
• I was given a letter to get further support from my GP and action plan should my GP not be willing to help
• I was given 100% attention.
• The clinician had clearly looked at my pre-meet questionnaire.
• I was given lots of reassurance and guidance
• I felt confident in the clinician’s knowledge and expertise.
• I received good advice with signposting to online resources which I accessed after the consult.
• I felt the issues I had been dealing with taken seriously and for the first time felt there could be a more differentiated approach for me.
• Have spent 4 years with many debilitating symptoms of the menopause. have visited doctors and A&E on many occasions and not one person has made any association between my symptoms and the menopause.
• The consultant that I spoke to actually listened to me!
• The consultation was extremely accessible, informative and within one week of the consultation I had been prescribed treatment by my GP
• I was very happy with the service and being able to talk to a specialist in the Menopause’
• I have not had much support or help from my GP and have struggled alone until now’
• I found this very helpful’
• It was so helpful, I was listened to and found help in finding the right HRT recommendation. There was also reassurance that if my GP did not help then I could be referred’
• I have suffered for a long, long time and found really needed this help. I am already seeing a difference a couple of weeks later. Thank you’
• Menopause hit me like a sledge hammer and accessing information has been quite confusing. I just wanted straight, clear, no nonsense information and that's what I got. Now I can make better informed decisions’
• Much needed and great to see the Trust pioneering this for staff health and wellbeing’
• Nice to know that the trust is taking note of something we have no control over but can affect us so badly – thank you’
• ‘Really helpful and reassuring, thank you, I am very grateful to have had access to this service’
• I had already consulted a GP about my symptoms but found the experience unhelpful and so I did not return and continued to suffer symptoms. This innovative pilot service was so accessible and easy to use and I feel so much happier to have started treatment’

With regards to the staff who ran and organised the clinic – when asked to reflect on the interactions/consultations they had with GSTT staff attendees during this pilot it was 9.8. The staff felt that the pre-questionnaire helped to focus the consultation. The clinic was very rewarding for the staff who ran it is as there was “Ad hoc” positive feedback from participants. Positive comments were spontaneously sent directly to Head of Nursing. Very positive feedback at team meetings/training day presentations of the “Show we care” innovation.

When asked what went well:

(1) the booking system
(2) asking the patients to fill in the form before attending.
(3) Chasing individuals who had not submitted self-referral forms
(4) sending across clinic diaries and forms the day before the clinic to the clinicians.

When asked what could be improved

(1) Send self-referral form over further in advance – cancel appointments if not sent in 48 h before appointment and put someone in from the waiting list.
(2) More lead time of clinic schedules to clinicians.
(3) Planned follow up survey post appointment to gauge effectiveness of advice and guidance leading to change/starting of treatment with GP
(4) Longer appointment time – 15 min for consult and five to do administration
(5) OH to send follow up letters and info out instead of clinicians
(6) Variation in timings of clinics to accommodate shift working

Main themes that emerged from feedback from attendees and staff generally about menopause care:

(1) Lack of support from and access to GPs.
(2) Low level of expertise amongst GPs.
(3) Lack of knowledge as to where to get reliable information and guidance.

## Conclusions

This innovative clinic for our menopausal staff has been very successful as assessed by patients (staff clinic attenders) and the staff doing the clinics. The plan now is for a fully funded clinic staffed by menopause specialists who currently run the clinics at GSTT or specialists who have been trained in our clinic. We also plan to do an audit of the staff in this pilot study in 6 months to find out how they are and to ascertain what lasting impact this had on their quality of life and working life.

We hope that this provides a useful template for other trusts to adopt so that our peri and postmenopausal workforce can be retained, not only in the NHS but in the wider female workforce.

